# Supplementary material for: Transparency of COVID-19-related research: A meta-research study
Source: PLoS One. 2023 Jul 26;18(7):e0288406. doi: 10.1371/journal.pone.0288406 (PMC10370694; doi:10.1371/journal.pone.0288406)
Supplement: S1 Text — (DOCX) [file pone.0288406.s001.docx]

## **S1 Text - Deviations from the protocol**

In the early protocol, we tried to catch COVID-19-related papers by searching COVID-19-related keywords in the title, keywords, and results of the open access papers. While this method managed to identify correct COVID-19-related papers 91% of the time, we decided to use the LitCovid database to have a more accurate estimate of true COVID-19-related papers.

Furthermore, to detect paper types, we first aimed to develop a code in R. As the majority of the papers were observational studies and letters, the accuracy was not optimal (about 65%). Thus, we changed the strategy and used the iloveevidence.com database of COVID-19 RCTs. To detect research articles and reviews, we did not change anything and simply used labels from the EPMC database.
