# Supplementary material for: Transparency of COVID-19-related research: A meta-research study
Source: PLoS One. 2023 Jul 26;18(7):e0288406. doi: 10.1371/journal.pone.0288406 (PMC10370694; doi:10.1371/journal.pone.0288406)
Supplement: S1 Table — (DOCX) [file pone.0288406.s002.docx]

## **S2 Table**

**S2 Table.** Citations to article and journal impact factor by transparency practices for RCTs.

| **Measurement** | **Citations to article** | | | **Journal Impact Factor (JIF)** | | |
| --- | --- | --- | --- | --- | --- | --- |
|  | Median (IQR) | | P-value | Median (IQR) | | P-value |
|  | With | Without |  | With | Without |  |
| COI disclosure | 2 (11) | 2 (11) | 0.447 | 4.5 (5.8) | 7.6 (5.0) | 0.199 |
| Funding disclosure | 2 (11) | 1 (7) | 0.105 | 4.6 (5.8) | 4.9 (5.4) | 0.505 |
| Protocol registration | 3 (18) | 1 (6) | <0.001 | 5.3 (13.0) | 4.0 (3.5) | <0.001 |
| Data sharing | 1 (7) | 2 (11) | 0.04 | 3.7 (3.8) | 4.7 (5.8) | 0.035 |
| Code sharing | 1 (7) | 2 (11) | 0.572 | 4.0 (5.1) | 4.6 (5.8) | 0.615 |

P-value based on the Wilcoxon rank sum test. 183 articles were published in journals with no impact factor. COI: conflict of interest; IQR: inter-quartile range.
