# Supplementary material for: Transparency of COVID-19-related research: A meta-research study
Source: PLoS One. 2023 Jul 26;18(7):e0288406. doi: 10.1371/journal.pone.0288406 (PMC10370694; doi:10.1371/journal.pone.0288406)
Supplement: S2 Table — (DOCX) [file pone.0288406.s003.docx]

## **S3 Table**

**S3 Table.** Citations to article and journal impact factor by transparency practices for reviews.

| **Measurement** | **Citations to article** | | | **Journal Impact Factor (JIF)** | | |
| --- | --- | --- | --- | --- | --- | --- |
|  | Median (IQR) | | P-value | Median (IQR) | | P-value |
|  | With | Without |  | With | Without |  |
| COI disclosure | 3 (10) | 4 (14) | <0.001 | 4.4 (2.6) | 4.4 (4.5) | 0.749 |
| Funding disclosure | 3 (10) | 3 (11) | 0.015 | 4.6 (2.8) | 4.0 (3.0) | <0.001 |
| Protocol registration | 2 (7) | 3 (10) | <0.001 | 3.5 (2.5) | 4.5 (2.8) | <0.001 |
| Data sharing | 3 (16) | 3 (10) | 0.001 | 4.9 (2.8) | 4.4 (2.8) | 0.033 |
| Code sharing | 3 (8) | 3 (10) | 0.634 | 4.9 (3.1) | 4.4 (2.8) | 0.406 |

P-value based on the Wilcoxon rank sum test. 4545 articles were published in journals with no impact factor. COI: conflict of interest; IQR: inter-quartile range.
