# Supplementary material for: Transparency of COVID-19-related research: A meta-research study
Source: PLoS One. 2023 Jul 26;18(7):e0288406. doi: 10.1371/journal.pone.0288406 (PMC10370694; doi:10.1371/journal.pone.0288406)
Supplement: S3 Table — (DOCX) [file pone.0288406.s004.docx]

## **S4 Table**

**S4 Table.** SCImago Journal Rank and H-index by transparency practices for RCTs.

| **Measurement** | **SCImago Journal Rank (SJR)** | | | **Journal H-index** | | |
| --- | --- | --- | --- | --- | --- | --- |
|  | Median (IQR) | | P-value | Median (IQR) | | P-value |
|  | With | Without |  | With | Without |  |
| COI disclosure | 1.3 (1.7) | 2.1 (2.2) | 0.019 | 110 (152.2) | 107 (209) | 0.965 |
| Funding disclosure | 1.3 (1.7) | 1.3 (2.5) | 0.157 | 110 (162) | 91 (146.5) | 0.117 |
| Protocol registration | 1.4 (3) | 1.2 (1.1) | <0.001 | 113 (163.8) | 104 (136) | 0.153 |
| Data sharing | 1.2 (1.1) | 1.3 (1.9) | 0.822 | 113 (285.5) | 110 (149) | 0.161 |
| Code sharing | 1.3 (2.1) | 1.3 (1.8) | 0.730 | 122 (100.2) | 110 (165) | 0.882 |

P-value based on the Wilcoxon rank sum test. 183 articles were published in journals with no impact factor. COI: conflict of interest; SD: standard deviation; IQR: inter-quartile range.
