# Supplementary material for: Transparency of COVID-19-related research: A meta-research study
Source: PLoS One. 2023 Jul 26;18(7):e0288406. doi: 10.1371/journal.pone.0288406 (PMC10370694; doi:10.1371/journal.pone.0288406)
Supplement: S4 Table — (DOCX) [file pone.0288406.s005.docx]

## **S5 Table**

**S5 Table.** SCImago Journal Rank and H-index by transparency practices for reviews.

| **Measurement** | **SCImago Journal Rank (SJR)** | | | **Journal H-index** | | |
| --- | --- | --- | --- | --- | --- | --- |
|  | Median (IQR) | | P-value | Median (IQR) | | P-value |
|  | With | Without |  | With | Without |  |
| COI disclosure | 1.1 (0.9) | 1.1 (1.5) | 0.349 | 71 (80) | 89 (103) | <0.001 |
| Funding disclosure | 1.2 (1) | 0.9 (0.9) | <0.001 | 75 (86) | 69 (78) | 0.016 |
| Protocol registration | 1 (0.8) | 1.1 (0.9) | <0.001 | 67 (84) | 73 (85) | <0.001 |
| Data sharing | 1.2 (1.1) | 1.1 (0.9) | <0.001 | 94 (86) | 72 (85) | <0.001 |
| Code sharing | 1.2 (1.3) | 1.1 (0.9) | 0.641 | 67.5 (75.5) | 73 (84) | 0.823 |

P-value based on the Wilcoxon rank sum test. 183 articles were published in journals with no impact factor. COI: conflict of interest; SD: standard deviation; IQR: inter-quartile range.
