# Supplementary material for: Transparency of COVID-19-related research: A meta-research study
Source: PLoS One. 2023 Jul 26;18(7):e0288406. doi: 10.1371/journal.pone.0288406 (PMC10370694; doi:10.1371/journal.pone.0288406)
Supplement: Attachment — Submitted filename: PONE-D-22-33929-Revision E.docx [file pone.0288406.s007.docx]

**PONE-D-22-33929**

**Transparency of COVID-19-related research: A meta-research study**

This is a very descriptive study in which the results of other studies are commented on without a clear relationship with the study addressed in this work.

Method

The authors rely on the results of the Europe PubMed Central database. Explain the reasons why they have chosen this source over others, as well as the possible biases that this may have produced.

Please, explain how do you measure the data sharing variable?

Describe what the tool used considers “valid” or considers dataset. Explain what the tool consists of. It is not enough just to cite it in the bibliography. For example, does it include supplementary material or just datasets?

Results.

In this chapter it is striking that nothing is said about the journals in Table 3 and the publishers in Table 4.

Table 5 only includes citations and impact factor. Why is the h-index and the number of articles published in open access not included?

Discussion

The discussion is poor in arguments and repeats again some of the results of the study. It seems to be unfinished or halfway through.

Some aspects to be considered and argued have to do with transparency, since transparency alone is not synonymous with quality.

Authors should at least relate transparency practices to other indicators of article quality, such as the number of citations received, impact factor, journal citation Indicator and quartile, since, on the other hand, it has been mentioned in the method.

The fact that review articles receive less funding is obvious, since this type of work is not usually funded, as they are not research work sensu stricto.

What are the implications of the fact that "COI disclosures seemed to be more common in articles published in lower-impact journals?

Why "adherence to transparent practices was at a similar level in COVID-19-related literature than in other biomedical literature analyzed with the same methods"?

References

What do you mean by "Scientometrics. 2021 kesäkuu"?
