# Supplementary material for: Transparency of COVID-19-related research: A meta-research study
Source: PLoS One. 2023 Jul 26;18(7):e0288406. doi: 10.1371/journal.pone.0288406 (PMC10370694; doi:10.1371/journal.pone.0288406)
Supplement: Attachment — Submitted filename: Response to reviewers_PLOS ONE.docx [file pone.0288406.s008.docx]

Associate Editor comments:

- The introduction should better explain open science and open data scenario with data sharing practices (data deposited in repositories or data from supplementary material, etc.), as well as code sharing. It is about transparency but no comments on FAIR principles and research data are done even in discussion.

Authors: Thanks for your comment. We revised the whole introduction part, and it now includes all the topics that you mentioned.

- Related to the methods: Document search strategy needs better description. As I understood you have searched for Covid-19 publications in PubMed database. At this point which are the terms used for the search equation?

-Also you have used LitCovid for searching articles and reviews and L.OVE platform for searching clinical trials, is that correct? With the merged PMIDs that you have obtained from the PubMed, LitCovid and L.OVE databases, a second search has been performed in Europe PubMed Central to obtain the full texts of the papers (can be difficult to understand even reading S1 text-deviations from the protocol).

Subsequently an analysis of the total papers obtained from EPMC has been done for 5 transparency practices (and results are showed by type of document: research article, review or RCT). All this search strategy should be summarized at the beginning of the results to improve readers' understanding. Even a flow chart with the data obtained (database and number or results) would be interesting and would facilitate understanding.

- The authors need to discuss their choice of database--and the fact that they only searched one database--as a limitation of their research. Why EPMC? Why not PMC for example?

Authors: Thanks for your valuable comment. We revised the Methods and the Results sections accordingly and added a Venn diagram and a flow diagram. In summary, we first searched for all the papers indexed in PubMed via Europe PMC (EPMC) by just specifying date (FIRST_PDATE:[2020-01-01 TO 2022-06-09]), the database of interest (SRC:"MED"), open-access status (OPEN_ACCESS:y), and English language (LANG:"eng" OR LANG:"en" OR LANG:"us") without any keywords. The EPMC includes all the articles indexed in several databases, including PubMed and PubMed Central (PMC). We chose EPMC because it has capabilities to be used for Open Science research (e.g., *europepmc* package in R). More information is available here: <https://europepmc.org/About>.

After catching over 1.5 million articles, we used the LitCovid database to check which of these articles were COVID-19-related. PubMed (and hence, EPMC) have filters for recognizing publication types (such as research articles, letters, reviews, RCTs, observational studies, etc.). However, based on our investigation, only research article and review labels are accurate, and many RCTs and observational studies are labelled solely as research articles rather than RCTs or observational studies. Therefore, we used the L.OVE database to detect COVID-19-related RCTs. Unfortunately, we did not find any database for detecting observational studies.

In the methodology, the tool that has been used should be better explained, because the manual validation that we can see in the supplementary excel is only for 100 documents.

Authors: Thanks for your comment. We revised the Methods section with complementary information in this regard. This tool has been previously validated for all biomedical research papers (<https://doi.org/10.1371/journal.pbio.3001107>). We performed the validation exercise to investigate whether this tool is still valid for COVID-19-related papers.

-Table 5 and supplementary tables needs to clarify. It is needed an explanation regarding the significance of Median (IQR) with/without.

Authors: Thanks for your comments. We added a thorough explanation and also moved Table S4 to the main text as Table 6 since we thought it also could add more insights to our results.

- In addition to the questions previously asked, as suggested by the 3 reviewers, please respond to each point that they have questioned.

As far as I can see, I cannot promise acceptance, but I would reconsider an improved version of the current manuscript.

Authors: Thanks for all your valuable comments. We think your comments and those of the reviewers improved our manuscript substantially.

Reviewer 1

The subject matter of the work presented is of great importance to the scientific community. Transparency is one of the fundamental pillars on which most emphasis is currently being placed by international organizations and funding agencies.

In my opinion, I believe that the article is interesting and useful for the scientific community.

However, it has some shortcomings that I believe should be addressed.

1. The paper mentions that the five practices that underpin transparency are data sharing, code sharing, COI disclosures, and protocol registration. Considering that not everyone is familiar with the concepts, I think a brief description of each in the enumeration of the concepts in the "Data extraction and synthesis" section of the methodology would be very useful. Based on this description, I believe that it should be further specified what is to be considered in this study for each element. This is particularly important in the case of data sharing since within this group it is not differentiated whether it is raw data or already processed material, as can be seen in the S7 Appendix for example in the open data statement of record 103787, which is not raw data.

Authors: Thanks for your valuable comment. We amended the methods section accordingly, with examples for more elaboration.

2. On the other hand, I think that, in addition to documentary typology, it would be very interesting to add some information on the distribution at least by country or by discipline, as I think it would give a richer context to the study. The subject matter of COVID-19 is very broad and the disciplines that deal with it in one way or another are very diverse. It would be very useful to know which disciplines are carrying out transparency practices and how, for example, which ones are sharing more code, or which ones are sharing more data. This type of information would enrich the discussion, and more robust conclusions could be drawn, since as they stand they are quite generic and highlight aspects that are not novel. In other words, we already know that more transparency is crucial to ensure credibility and that society should benefit from it, but at this point, it would be interesting to sharpen the focus and find out, within the medical disciplines, who does what and how, since genetics and its tradition of sharing data (regardless of covid) have nothing to do with more clinical disciplines.

Authors: Thanks for your thoughtful insight. We agree that this is very important. However, do not have the data for countries or discipline. In fact, we aim to do so in our next project for all 59 categories of clinical medicine. For such a research, we should have a list of journals in each filed of health and medicine and the countries of the authors. Unfortunately, Europe PMC and PubMed do not share such data when returning the search results. Therefore, some alternative methods such as using categorization of the other databases (e.g., JCR or SJR) should be used at the first stage (searching for the articles). To sum up, we have constraints to do so with the current research.

Reviewer 2

This is a very descriptive study in which the results of other studies are commented on without a clear relationship with the study addressed in this work.

Method

1.The authors rely on the results of the Europe PubMed Central database. Explain the reasons why they have chosen this source over others, as well as the possible biases that this may have produced.

Authors: Thanks for your comment. MEDLINE (and hence, PubMed) is the most important database of articles published in health and medical journals. The Europe PMC (EPMC) has exactly the same database as MEDLINE and PubMed. As they have mentioned in their About page (<https://europepmc.org/About>), it includes PubMed and PubMed Central (PMC) and more than 30 other sources. In this research, we just used the PubMed part of it. The reason for using EPMC was that it enabled us to do all the works automatically which PubMed website is not capable of doing so. The results of the search queries in EPMC and PubMed are exactly the same and, therefore, we can tell that we used PubMed (or MEDLINE) through EPMC (just like using MEDLINE through Ovid).

We added a sentence for the clarification under the “Data sources and study selection” section of the Methods which reads:

“This database includes all the records available through PubMed and PubMed Central and also enabled us to retrieve the record automatically which is not available through PubMed website.”

2.Please, explain how do you measure the data sharing variable?

Authors: Thanks for the comment. We amended the “Data extraction and synthesis” section accordingly. Now it reads:

“We used a validated and automated tool developed by Serghiou et al. (6) suitable to identify these five transparent practices from articles in XML format from the EPMC database. Briefly, this tool uses some keywords to identify adherence to each indicator using regular expressions. For example, it searches for phrases commonly associated with a COI disclosure (e.g., “conflicts of interest,” “competing interests,” “Nothing to disclose.,” etc.) in the body or titles of the sections of the text file of an article. For data and code sharing detection, it both detects shared as a supplement or shared in a general (e.g., figshare, OSF, GitHub, etc.) or field-specific repository (e.g., dbSNP, ProteomeXchange, GenomeRNAi, etc.) as adherence to transparency to data/code sharing. However, those articles that indicated "data available under request" would be classified as no data available, since it is unlikely to obtain these data (11). Overall, all the mentions of the indicators were classified as affirmative when the article explicitly contained it.”

3.Describe what the tool used considers “valid” or considers dataset. Explain what the tool consists of. It is not enough just to cite it in the bibliography. For example, does it include supplementary material or just datasets?

Authors: Thanks for the comment. As we mentioned above, we elaborated more on the algorithm of the tool.

Results.

4.In this chapter it is striking that nothing is said about the journals in Table 3 and the publishers in Table 4.

Authors: Thanks for your keen comment. We aimed to just include the most important aspects of these tables (such as “55% of research articles published in PLoS One had shared data”). However, we extended this paragraph to show more interesting findings of these Tables. Now it reads:

“There were journal and publisher-related differences in all five practices (Tables 3 and 4). All the papers from MDPI and PLoS had COI and funding disclosure. Whereas COI and funding disclosure was available for almost all the RCTs published in the New England Journal of Medicine and The Lancet, they rarely shared their data (0% and 2.9%, respectively). The highest percentage of data sharing was found in PLoS One research articles (54.8%) and RCTs (70.0%). RCTs published in The Lancet and The Lancet Respiratory Medicine had the highest adherence to protocol registration, with 94.1% and 100%, respectively. Code sharing was a rare practice, with Scientific Reports (14.2%) and PLoS (11.1%) be the top journal and publisher for adherence in this practice. None of the journals with the highest number of RCTs in the sample shared their codes.”

5.Table 5 only includes citations and impact factor. Why is the h-index and the number of articles published in open access not included?

Authors: Thanks for your comment. We transferred Table 6 from Appendix S4 to the main text to show the results for H-index and SCImago Journal Rank (SJR). We also added the following paragraph:

“Table 6 illustrates the median SCImago Journal Rank (SJR) and H-index for articles with and without adherence to each transparency indicator. Research articles that adhered to each of the five indicators had higher median SJR and H-index. The results for RCTs and reviews are available in Tables S4 and S5.”

Discussion

6.The discussion is poor in arguments and repeats again some of the results of the study. It seems to be unfinished or halfway through.

Authors: Thanks for the comment. We extended the discussion and amended it considerably.

7.Some aspects to be considered and argued have to do with transparency, since transparency alone is not synonymous with quality.

Authors should at least relate transparency practices to other indicators of article quality, such as the number of citations received, impact factor, journal citation Indicator and quartile, since, on the other hand, it has been mentioned in the method.

Authors: Thanks for your valuable comment. We had mentioned article quality indices in the discussion very briefly: “differences in transparent practices according to received citations and JIF were small”. We elaborated on this in a paragraph, as below:

“Whereas transparency is deemed to be important for health research (PMID: 26821973), when we compared the level of transparency for each domain based on some indices related to the quality of the papers and journals (Tables 5 and 6), we found inconsistent results. For example, there were little differences between articles adhered to transparency indicators in terms of citation. The same was seen for SJR index. However, journals with higher JIF and H-index had better adherence to transparency indicators. This may imply that publishers pay attention to the trends and have updated their policy regarding transparency, especially in COI and funding disclosure. However, researchers cite papers without considering their level of transparency.”

8.The fact that review articles receive less funding is obvious, since this type of work is not usually funded, as they are not research work sensu stricto.

Authors: Thanks for your keen insight. In fact, the tool that we used just determines mentioning of funding disclosure and does not differentiate between articles that received funding with those which did not. In other words, if there was a sentence such as “no funding” in the paper, that paper will be considered as transparent in terms of funding disclosure. However, for clearance, we added examples in the “Data extraction and synthesis” section of the Methods.

Furthermore, the adherence of reviews to transparency indicators is comparable to research articles as depicted in Table 1).

9.What are the implications of the fact that "COI disclosures seemed to be more common in articles published in lower-impact journals?

Authors: Thanks for the comment. That sentence was not accurate based on Table 5. We amended it as below:

“In addition, journals with higher JIF seemed to be more transparent, except for the COI disclosures”.

Other explanations are available in the paragraph added for your 7th comment.

10.Why "adherence to transparent practices was at a similar level in COVID-19-related literature than in other biomedical literature analyzed with the same methods"?

Authors: Thanks for the comment. We added two sentence to the end of that paragraph for possible reasons:

“Furthermore, any initiative and movement needs time to be effective. Another reason could be the fact that these changes should be implemented by publishers and journals.”

References

What do you mean by "Scientometrics. 2021 kesäkuu"?

Authors: Thanks for your keen comment. This is Finnish for “June”. We substituted it with “June”.

Reviewer 3

This article deals with the concept of transparency in works (research, RCT, reviews) published in PubMed indexed journals about COVID-19.

My major concerns focus the lack of explanations.

1. The background section is too short, and it is excessively centered in transparency, omitting that concepts like data sharing go beyond transparency. Authors do not explain what is data sharing or code sharing and why they are so important for transparency, among other considerations.

Authors: Thanks for your comment. This concern was also raised by other reviewers. We amended all the comments regarding the Background.

2.The Methods section lacks of an explanation about why Europe PMC repository was selected, despite of the potential problems about this decision were included in the Discussion section.

Authors: Thanks for the comment. MEDLINE (and hence, PubMed) is the most important database of articles published in health and medical journals. The Europe PMC (EPMC) has exactly the same database as MEDLINE and PubMed. As they have mentioned in their About page (<https://europepmc.org/About>), it includes PubMed and PubMed Central (PMC) and more than 30 other sources. In this research, we just used the PubMed part of it. The reason for using EPMC was that it enabled us to do all the works automatically which PubMed website is not capable of doing so. The results of the search queries in EPMC and PubMed are exactly the same and, therefore, we can tell that we used PubMed (or MEDLINE) through EPMC (just like using MEDLINE through Ovid).

We added a sentence for the clarification under the “Data sources and study selection” section of the Methods which reads:

“This database includes all the records available through PubMed and PubMed Central and also enabled us to retrieve the record automatically which is not available through PubMed website.”

We also mentioned the limitations of this decision in the discussion.

3.Table 5 is not sufficiently understandable.

Authors: Thanks for your comment. We added more details for that part and also added Table 6 which includes SCImago Journal Rank (SJR) and H-index. Now it reads:

“Apart from COI disclosure, research articles that adhered to transparency practices were published in journals with higher median JIF. Research articles with data or code sharing received one more median citation than those without data or code sharing. In contrast, articles without COI or funding disclosure received one more median citation compared to those which adhered to these indicators (Table 5). Tables S2 and S3 show the results for RCTs and reviews.”

“Table 6 illustrates the median SCImago Journal Rank (SJR) and H-index for articles with and without adherence to each transparency indicator. Research articles that adhered to each of the five indicators had higher median SJR and H-index. The results for RCTs and reviews are available in Tables S4 and S5.”
