# Supplementary material for: Transparency of COVID-19-related research: A meta-research study
Source: PLoS One. 2023 Jul 26;18(7):e0288406. doi: 10.1371/journal.pone.0288406 (PMC10370694; doi:10.1371/journal.pone.0288406)
Supplement: Attachment — Submitted filename: Response to reviewers_PLOS ONE.docx [file pone.0288406.s009.docx]

Journal Requirements:

Please review your reference list to ensure that it is complete and correct. If you have cited papers that have been retracted, please include the rationale for doing so in the manuscript text, or remove these references and replace them with relevant current references. Any changes to the reference list should be mentioned in the rebuttal letter that accompanies your revised manuscript. If you need to cite a retracted article, indicate the article’s retracted status in the References list and also include a citation and full reference for the retraction notice.

Authors: We reviewed all the references.

**Additional Editor Comments:**

The paper has been improved following the recommendations of the academic editor and reviewers. It is important for the acceptation of the manuscript that the minor changes suggested will be taken into account.

1. Background. There are spaces in the text before citations 3 and 4 that need to be removed.

Authors: Thanks for your keen look. We removed these spaces and also some others. We used Google Docs for the previous revision, and that caused some deformities in the text. For this revision, we used Microsoft Word, and we hope it works fine this time.

2. Background. The sentence: To facilitate rapid and collaborative scientific research, over 100 organizations…please could authors insert the reference to support this sentence.

Authors: We added the pertaining reference which is a press release from Wellcome Trust: <https://wellcome.org/press-release/sharing-research-data-and-findings-relevant-novel-coronavirus-ncov-outbreak>

3. Methods. At the end of Data sources and study selection it is mentioned: As we want to focus solely on RCTs that have been done, we applied two filters: “RCT” and “Reporting data,” on the L·OVE website and then downloaded the dataset with these characteristics. Again, we used PMIDs to merge datasets. Please, check if is correct that sentence? The PMIDs are used to recover the articles?, not datasets. In fact, authors are explaining in the next paragraph of Data extraction and synthesis: we assessed articles’ adherence to five transparent practices.

Authors: Thanks for the comment. We clarified the sentence. Now it reads:

“We used PMIDs of COVID-19-related RCTs provided in this downloaded dataset from the L·OVE website to detect RCTs in our main dataset of all open-access COVID-19-related papers.”.

More clarification: The LOVE website allows to download a dataset of articles with desired characteristics (COVID-19-related RCTs in our case). Then, we used the intersection between this dataset and our dataset of all COVID-19-related papers to detect open-access COVID-19-related RCTs.

4. Methods. Regarding journal and article information in METHODS, please explain what IQM is and how is calculated. It appears in the tables of results many times.

Authors: Thanks for the comment. We clarified it in the last paragraph of methods:

“We used the interquartile range (the third quartile (Q3) – the first quartile (Q1)) when the data were skewed.”.

5. Methods. In the table 5 Where did the citations come from? Are calculated through the Web of Science, since is the same table of JIF. Explain please. To clarify, authors may include citations and Impact index of the journals were obtained from JCR. Which edition of JCR are you using? Mainly because for the other impact indicators in the following table you are using SJR and h-index from another database (SJR).

Authors: Thanks for the comment. As we have indicated in the last paragraph of the “Data extraction and synthesis”, it comes from PubMed (through EPMC database):

“Basic journal- and article-related information (publisher, publication year, citations to article and journal name) were retrieved from the EPMC database.”

Also, in the same paragraph, the source of JIF is indicated:

“The impact index of the journals (JIFs) was obtained from the Journal Citation Reports 2021 (jcr.clarivate.com) and the SCImago Journal Rank 2020 (SJR) and H-index indicators from the SCImago website (scimagojr.com).”

We added the editions to the text (2021 and 2020, respectively).

6. Methods. Data analysis. Regarding the tables of results, appears in the legend of the tables SD: standard deviation. But never appears this acronym in the tables, so this may be confusing.

Authors: Thanks for your keen comment. We removed the SD.

7. Results. Transparency practices. It appears figure 1, however authos have to correct writing figure 3, as well as Figure 3-right.

Authors: Thanks for your comment. We carefully revised all the figures and legends and references to them in the text.

8. Results. At the end of table 4: Tables S2 and S3 show the results for RCTs and reviews.
I can not see this supplementary tables. I only can see Table S4, Table S5 and S6 appendix in the submission files.

Authors: We think there has been some mistake when submitting the previous revision. Now you should receive Tables S2 and S3 files.

9. Results. Table 5. SD appears in the explanatory legend below the table: standard deviation. But this acronym does not appear in the table. For example, IQR appears in the table, and it is explained as inter quartile range. At this point, please explain the meaning/calculation of Median inter-quartile range (IQR) in methodology.

Authors: Thanks for the comment. As we mentioned in the response for comments 4 and 6, we added the explanation for IQR and removed SD from all the tables.

10. Results. Of 100 random articles for validation, 20 discrepancies (out of 500, 4%), could authors explain the meaning of: out of 500, 4%.

Authors: Thanks for the comment. Because every articles is being assessed for 5 indicators, we will have 5x100=500 indicators overall. Of these, 20 (4%) had discrepancies. We added “five indicators per article” The paragraph now reads:

“Of 100 random articles for validation, 20 discrepancies (out of 500 (five indicators per article), 4%) between the automatic tool and manual checking were found: 9% for open data, 2% for open code, 1% for COI disclosure, 6% for funding disclosure, and 2% for registration (S7 Appendix).”

11. Results. (S7 Appendix). I cannot see S7 appendix.

Authors: We think there has been a mistake in submitting the appendices. We carefully checked that all the appendices are included in this submission.

12. Discussion. Due to the manuscript has many tables, it would be great if the authors could synthetize in brief short lines at the beginning of the discussion the results obtained.

Authors: Thanks for the suggestion. We added an extra summary at the beginning of the discussion. Now it reads:

“Our study showed that adherence to transparent practices increased in COVID-19-related medical literature from 2020 to 2022. Adherence to reporting COI disclosure was high throughout the study period. In addition, most articles had funding disclosure. Data sharing, code sharing and protocol registration were rare but improved little over the study period. Higher adherence to COI disclosure and protocol registration was seen in randomized trials and reviews than in other research articles. The majority of research papers and reviews adhered to two or fewer indicators, whereas the majority of RCTs adhered to three or fewer indicators. Only 53 papers adhered to all indicators, overall. Journal- and publisher-related differences in transparency practices were clear. While some journals and publishers were completely transparent in terms of COI and funding disclosure, they had poor performance for the other three indicators. In addition, journals with higher JIF and H-index seemed to be more transparent, except for the COI disclosures. Otherwise, differences in transparent practices according to received citations and JIF were small.”
